# Wellens Syndrome Corollaries: A Call for Definition with a Case Series

**DOI:** 10.5811/cpcem.35877

**Published:** 2025-06-08

**Authors:** Addison R. Sparks, Patrick M. Bruss

**Affiliations:** * The University of Toledo College of Medicine & Life Sciences, Department of Emergency Medicine, Toledo, Ohio; † Promedica Monroe Emergency Medicine Residency, Department of Emergency Medicine, Monroe, Michigan

**Keywords:** Wellens, electrocardiogram, ischemia, left anterior descending artery, acute coronary syndrome

## Abstract

**Introduction:**

First described in 1982, the Wellens wave is an electrocardiographic (ECG) finding indicative of a critical lesion of the left anterior descending artery. These T-wave findings are classically found in ECG leads V2 and V3, although they may extend into the lateral leads V4–V6.

**Case Series:**

We present three cases of patients with Wellens waves that were found only in leads V3 and V4 and did not include V2.

**Conclusion:**

We suggest that the classical definition of T-waves in leads V2 and V3 is not the only manifestation of Wellens waves to indicate pathology. Wellens waves found in two contiguous leads in leads V1–V6 can be considered Wellens corollaries, thereby requiring the same emergent treatment as classical Wellens syndrome. We also recognize the need for a consensus on the inclusion criteria of Wellens syndrome, particularly the laboratory and ECG findings that define the disease.

## INTRODUCTION

First described by Dutch cardiologist Hein JJ Wellens and colleagues in 1982,^1^ Wellens waves are subtle, difficult-to-detect changes present on electrocardiogram (ECG) as T-wave abnormalities during ventricular repolarization. They are classified as Wellens type A or B (1 or 2) according to the T-wave morphology.^2^ Type A (or 1) Wellens is less common and involves biphasic T-waves in the early precordial leads.^2^,^3^ Wellens syndrome more often presents with deeply inverted T-waves in these precordial leads, which is classified as Wellens type B (or 2).^2^,^3^ These T-waves can be present for as long as several weeks.^4^ The current definition requires that T-wave abnormalities be noted in precordial leads V2 and V3 but may also occur in V1, V4, V5, and V6.^1^,^2^,^5^ The depth of T-wave inversions required for diagnosis has not been defined in the literature. The finding of Wellens waves is a highly specific indication for a pre-infarction, proximal occlusion of the left anterior descending (LAD) coronary artery, which causes an acute infarction and life-threatening dysfunction of the left ventricle, necessitating urgent cardiac catheterization.^1^,^2^

It is important to note that ECGs are measured at one specific time point and can change over time. A Wellens-appearing ECG, as a pre-infarction state, is dynamic and can later instead meet ST-elevation myocardial infarction (STEMI) criteria. Interestingly, one cross-sectional study and one systematic review have noted the presence of Wellens changes in the ECGs of patients who did not have an LAD occlusion or had multivessel disease.^6^,^7^ Those studies found that several patients met the criteria of Wellens syndrome but did not have an LAD occlusion and instead had occlusions of the right coronary artery, left coronary artery, or left circumflex artery.^6^,^7^

Diagnosis of Wellens syndrome also includes recognition of a lack of serum marker abnormalities (from some sources), a lack of pathologic Q-waves, presence of a normal R-wave progression, and an ST-segment that is not highly elevated.^4^,^5^,^8^ It is noteworthy that there is not a consensus on whether cardiac biomarkers must be within normal limits to make the diagnosis, and the original description of Wellens syndrome did not comment on whether serum chemistry changes were a diagnostic requirement.^1^ However, troponin levels were not measured in the 1980s as they are today. Wellens and colleagues relied on creatinine phosphokinase, lactate dehydrogenase, and glutamic oxaloacetic transaminase.^1^ The criteria for diagnosing Wellens syndrome has evolved since that time, and consensus is now needed. Some sources say these biomarkers, such as troponin, can be minimally elevated in Wellens syndrome.^4^,^5^ A suitable threshold for cardiac biomarkers needs to be defined; otherwise, it is difficult to separate Wellens syndrome as an independent pathology from a non-STEMI.

Additionally, Wellens syndrome must not be confused with left ventricular hypertrophy or right bundle branch blocks, which can include repolarization abnormalities such as T-wave inversions.^4^ Poor R-wave progression is also an exclusion criterion of Wellens.^4^,^5^ It is also critical to note that Wellens changes have been reported as “Wellens variants” on an ECG secondary to coronary vasospasm, such as vasospastic angina or cocaine-induced vasospasm.^7^ In fact, the initial description of Wellens syndrome included one patient with Prinzmetal angina and initial Wellens ECG changes that normalized after treatment with a calcium antagonist.^1^ One report presented a case of potential Wellens syndrome in the setting of a known left-septal fascicular block, which is supplied by the LAD.^9^ However, the patient in that specific case was experiencing chest pain at the time of the ECG recording, which by definition excluded the definition of Wellens syndrome.

It is possible to have a history of anginal pain, but these ECG findings must be obtained from a pain-free episode to be considered Wellens syndrome.^4^,^10^ The initial description of Wellens syndrome included patients who had previously experienced chest pain but whose characteristic ECG changes were obtained during pain-free episodes.^1^ Electrocardiogram changes characteristic of Wellens syndrome can appear even after anginal pain resolution.^11^

The risk factors for Wellens reflect what is expected when discussing acute coronary syndromes; hypertension, diabetes, and family history were more prevalent in the Wellens cases.^6^ Interestingly, smoking and hyperlipidemia were less prevalent at 14.2% and 22.5%, respectively.^6^ However, it is difficult to draw conclusions from one small study, highlighting the need to investigate Wellens-specific risk factors. Another study found that Wellens patients were less likely to have a previous history of heart disease or vessel occlusion, extrapolating that Wellens is more prevalent as an initial presentation of cardiac disease.^12^

When this condition was first described, it was found that 75% of patients with this wave pattern who did not undergo surgical treatment developed an infarction of the anterior heart wall within a few weeks.^1^ These patients had non-diagnostic serum biomarkers and were initially treated with nitroglycerin and a calcium-channel blocker as their ECGs normalized over several days; unfortunately, they died later due to vessel occlusion. One study found that there was no significant difference in 24-month survival of Wellens patients compared to patients with non-Wellens acute coronary syndrome.^12^ Despite its critical implications, there is no consensus in the literature as to whether Wellens is considered a true STEMI or a “STEMI equivalent.”

*CPC-EM Capsule*

What do we already know about this clinical entity?

*We know the typical presentation of Wellens syndrome, including T-wave abnormalities in electrocardiogram leads V2 and V3 and lack of chest pain or cardiac biomarker elevation*.

What makes this presentation of disease reportable?

*The presence of T-wave abnormalities in leads V3 and V4, excluding V2, in patients that had near-complete left anterior descending artery blockage is an unusual presentation of Wellens syndrome*..

What is the major learning point?

*Wellens’ syndrome corollaries can be present and excellent clinical acumen is needed to diagnose. There is a need for clarification of Wellens’ diagnostic criteria*.

How might this improve emergency medicine practice?

*This may expand consideration of Wellens’ syndrome in forming a differential diagnosis and call for clarification and definition of the diagnostic criteria*.

While involvement of leads V2 and V3 is the classically accepted presentation of Wellens syndrome, we discuss below three cases of “Wellens corollaries” to consider Wellens waves in leads V3 and V4 that excluded V2. In each of the three cases, the patient with biphasic T-waves in leads V3 and V4 without V2 underwent cardiac catheterization, and prominent LAD stenosis was discovered.

## CASE SERIES

### Case One

A 45-year-old male presented to the emergency department (ED) with the chief complaint of back pain. The patient reported acute onset of pain while moving a water heater up a flight of stairs. He described the pain as dull in nature, constant, radiating through to the center of his chest, worse with exertion, and accompanied by nausea and shortness of breath. He had no past medical history and took no medications, nor did he have a family history of coronary artery disease.

**Image 1:**
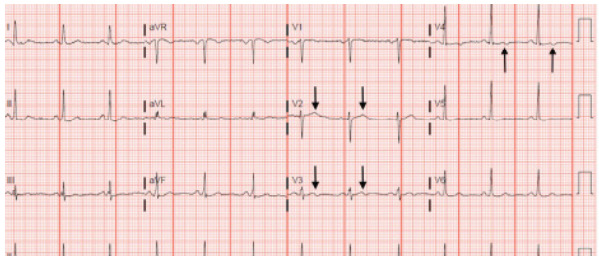
Concerning biphasic T-waves in leads V3 and V4, but not in V2 (arrows), which were present in a 45-year-old male with acute onset of back pain. Cardiac catheterization revealed 99% stenosis of the left anterior descending artery.

Physical exam revealed an inability to reproduce the pain with palpation. Workup included complete blood count (CBC), basic metabolic panel (BMP), troponin, D-dimer, and chest radiograph, which were all unremarkable. The patient had no pain in the ED and no symptoms while the ECG was conducted. The ECG was concerning for subtle biphasic T-waves in leads V3 and V4, with an unremarkable V2 (Image 1). This biphasic wave is initially positive and then trends negative. The absence of pain, presence of normal cardiac biomarkers, and ECG changes excluded STEMI or non-STEMI but fit the classic definition of Wellens syndrome. The patient was admitted to the hospital and had a stress test with positive results. He then underwent cardiac catheterization that revealed 99% stenosis of the LAD.

### Case Two

**Image 2:**
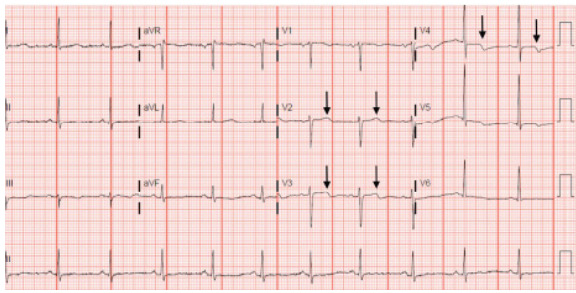
Biphasic T-waves in leads V3 and V4, but not in V2 (arrows), which were present in a 79-year-old female with a chief complaint of fatigue and several risk factors. Cardiac catheterization revealed 98% stenosis of the left anterior descending artery.

A 79-year-old female presented to the ED with a chief complaint of fatigue. She had multiple risk factors including hypertension, hyperlipidemia, and tobacco use. Her physical exam was unremarkable, and she denied any pain. Diagnostic workup included CBC, BMP, and chest radiograph, which were all within normal limits. The troponin was slightly elevated at 0.07 nanograms per milliliter (ng/mL) (reference range ≤0.04 mg/mL). The absence of ST-segment elevations excluded the diagnosis of STEMI; however, the elevated biomarker and lack of pain did not clearly indicate non-STEMI. The ECG was concerning for the presence of biphasic T-waves, isolated to V3 and V4 (Image 2). These T-wave inversions were subtle compared to lead V2. The patient was admitted to the hospital and underwent cardiac catheterization, which showed 98% stenosis of the LAD.

### Case Three

**Image 3:**
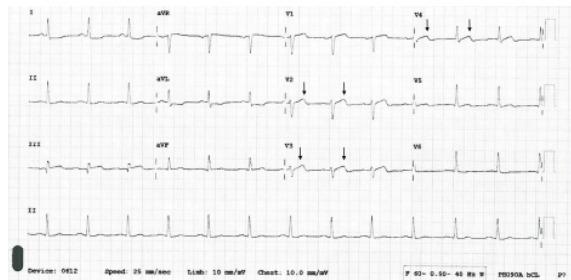
Electrocardiogram (ECG) leads V3 and V4 with biphasic T-waves are absent in V2 (arrows) in the ECG of a 54-year-old male with a chief complaint of chest pressure. The T-waves were initially positive and then trended negative. Cardiac catheterization revealed a 99% stenosis of the left anterior descending artery.

A 54-year-old male presented to the ED with a chief complaint of chest pressure. He had a family history of coronary artery disease, hypertension, and hyperlipidemia. He stated that the pain woke him up in the middle of the night but had resolved spontaneously prior to arrival. Physical exam was unremarkable, and he experienced no symptoms while the ECG was performed. Diagnostic CBC, BMP, troponin, and chest radiograph were all within normal limits. However, the ECG was concerning for biphasic T-waves in leads V3 and V4 that were absent from V2 (Image 3).

The different appearance of the T-waves in leads V2 and V3 were subtle but pertinent to the discussion. Importantly, the R-wave progression did not meet the classic definition of poor R-wave progression (R wave ≤3 millimeters), but the R-wave progression in this ECG appeared atypical and should have hinted at anterior infarction. Other features that possibly indicated infarction included the mild ST-elevation in lead III, ST-segment and T-wave deviation in the same direction in leads V3 and V4, and questionable ST-segment depression in the lateral leads. The absence of pain, the presence of normal cardiac biomarkers, and the ECG changes excluded STEMI or non-STEMI but did fit the classic definition of Wellens syndrome. The patient was admitted to the hospital, where serial troponin values were elevated. Catheterization discovered a 99% stenosis of the LAD.

## DISCUSSION

The classic definition of Wellens syndrome involves biphasic T-waves in ECG leads V2 and V3, while sometimes extending into leads V4, V5, and V6.^2^ This ECG finding is specific for stenosis of the proximal LAD.^2^ From the three cases presented, we propose considering Wellens corollaries when Wellens waves are noted in leads V3 and V4, even when not seen in lead V2. Future studies should evaluate whether these changes in any two contiguous leads could be considered Wellens syndrome. In the three cases discussed here each patient presented with biphasic T-waves in ECG leads V3 and V4, with a monophasic T-wave in V2. This falls outside the classical definition of Wellens syndrome; however, all three cases still necessitated emergency cardiac catheterization that found a dangerously stenotic LAD.

## LIMITATIONS

Subsequent ECGs were not available for the cases discussed above. It is also important to note that there are numerous findings on the ECG in Case 3 that should prompt suspicion for infarction.

## CONCLUSION

The three patients discussed above presented to the ED with various symptoms and histories. Electrocardiogram revealed biphasic T-waves (Wellens waves) in leads V3 and V4 but, notably, not in V2. While this presentation differs from the accepted definition of Wellens waves, these patients still qualified for cardiac catheterization and had significant stenosis of the left anterior descending artery. Therefore, we propose expanding the definition of Wellens wave/Wellens syndrome to include biphasic or inverted T-waves present in any two adjacent leads of V1–V6. We also recognize the need for clarification of the definition of Wellens criteria, particularly with respect to cardiac biomarkers and diseased vessel territory. There is also a need for further investigation into Wellens-specific risk factors and survival.
